# The relationship between online learning self-efficacy and learning engagement: the mediating role of achievement motivation and flow among registered nurses

**DOI:** 10.3389/fpsyg.2025.1629174

**Published:** 2025-09-26

**Authors:** Tong Zhou, Yinhai Chen, Xixi Li, Luyao Yang, Zhonglei Zhao

**Affiliations:** ^1^North Sichuan Medical College, Nanchong, China; ^2^Suining Central Hospital, Suining, China

**Keywords:** nursing education, online learning self-efficacy, learning engagement, mediation analysis, flow

## Abstract

**Introduction:**

Learning engagement is a critical predictor of core professional competencies in nursing, yet its influencing factors remain unclear. This study examined the mediating roles of achievement motivation and flow in the relationship between online learning self-efficacy and learning engagement among registered nurses (RNs).

**Methods:**

A cross-sectional electronic survey was conducted among 657 RNs from two general hospitals in Sichuan Province, China, between March and May 2024. Participants completed validated instruments, including the Adult Online Learning Self-Efficacy Scale, Achievement Motives Scale (AMS), Educational Flow Scale (EduFlow-2), and Learning Engagement Scale. Descriptive statistics, Pearson correlation, and mediation analysis were conducted using SPSS v29.0. Structural equation modeling was performed using AMOS v24.0.

**Results:**

The average learning engagement score was 61.55 ± 11.07. Online learning self-efficacy (*r* = 0.498, *p* < 0.01), achievement motivation (*r* = 0.550, *p* < 0.01), and flow (*r* = 0.424, *p* < 0.01) were all positively associated with learning engagement. Achievement motivation (22.2%), flow (24.7%), and their chain effect (11.2%) partially mediated the link between self-efficacy and engagement.

**Discussion:**

Online learning self-efficacy influences RN learning engagement both directly and indirectly, with mediation effects exerted through achievement motivation and flow. These findings highlight the importance of fostering self-efficacy and motivational processes to enhance engagement in online nursing education.

## 1 Introduction

In recent years, the global health sector has faced numerous challenges, including the persistent threat of infectious diseases, the rising prevalence of non-communicable diseases, and the growing aging population (World Health Organization, n.d.). These challenges demand a highly qualified nursing workforce to address increasingly complex health needs. However, the shortage of nursing personnel and skills gap remain significant global issues, with an estimated shortage of approximately 4.6 million registered nurses (RNs) worldwide by 2030 (World Health Organization, 2020). Lifelong learning and continuous professional development have become widely recognized as essential in the nursing field. Therefore, the effectiveness of nursing education, especially in the face of new clinical challenges, has become a key factor in improving the quality of nursing care.

Virtual simulation refers to the creation of virtual environments using computer-generated imagery, in combination with technologies such as virtual reality, augmented reality, and computer graphics, to replicate real or hypothetical scenarios (Plotzky et al., 2021). Through interaction with these environments, users gain an immersive experience that enables skill training and decision-making processes. In recent years, virtual simulation-based e-learning has emerged as an efficient and cost-effective tool for self-improvement and hospital training (Hao et al., 2022). Research indicates that high-quality virtual simulation learning helps improve nurses' professional skills (Ding et al., 2024), enhance communication abilities (Liaw et al., 2023), and increase their self-confidence and self-efficacy through repeated practice (Liu et al., 2023).

Learning engagement, a crucial measure of learning quality and training success, encompasses cognitive, emotional, and behavioral involvement (Alemayehu and Chen, 2023). The level of learning engagement significantly influences the long-term success of RNs (Mohamed Bayoumy and Alsayed, 2021), as it helps enhance their knowledge and practical skills, problem-solving abilities, and fosters independent learning and critical thinking. Xu et al. (2023) emphasized that emotional engagement effectively promotes learning outcomes in basic medical knowledge and clinical skills, with the combination of emotional and cognitive engagement serving as a reliable predictor of medical knowledge learning outcomes. These competencies are directly linked to the quality and safety of patient care. However, learners often experience low levels of engagement due to distractions and procrastination (Alemayehu and Chen, 2023). Therefore, understanding the antecedents of learning engagement among RNs is critical for improving clinical nursing practice and enhancing overall service quality.

Social Cognitive Theory (SCT) posits that an individual's behavior and choices are significantly influenced by self-efficacy (Bandura, 2001). Learners with high self-efficacy are more confident when facing challenges and tend to demonstrate stronger engagement in their learning. Several studies have confirmed that self-efficacy is a critical psychological factor influencing learning engagement (Heo et al., 2022; Pan, 2022). Although relevant studies have established the relationship between self-efficacy and learning engagement, they primarily focus on traditional face-to-face learning environments or or student populations (Alrashidi and Alshammari, 2024; Zou et al., 2024), without fully considering the unique demands of online learning in high-pressure professional settings, such as in nursing. Specifically, self-efficacy in virtual simulation environments differs from that in traditional settings, primarily due to enhanced autonomy, reduced social presence, and a higher risk of disconnection (Jiang et al., 2025). It is essential to identify new factors and moderators to better explain the dynamic nature of RNs' online learning self-efficacy and learning engagement. A meta-analysis revealed that achievement motivation (an intrinsic driver of learners) and flow (the core mechanism of immersion in virtual simulation) are both associated with learning engagement (Zhang et al., 2021). Given the unique nature of virtual simulation learning environments and the self-driven characteristics of nursing professionals, this study focuses on examining the roles of achievement motivation and flow as key variables.

To address the aforementioned gap, this study will investigate the relationship and mediating mechanisms between online learning self-efficacy and learning engagement among RNs in a virtual simulation-based learning environment. The findings will provide theoretical insights for nursing managers to improve training programs, enhance RNs' learning outcomes, and clinical practice capabilities, thereby contributing to the healthy development of the nursing workforce.

## 2 Literature review

To explore the influencing mechanisms of online learning engagement among registered nurses, this review focuses on three key psychological constructs: online learning self-efficacy, achievement motivation, and flow experience.

Self-efficacy refers to an individual's confidence and belief in their ability to successfully complete a specific task or achieve a goal (Wang et al., 2021). In the context of online learning, Pituch and Lee (2006) define online learning self-efficacy as the learner's confidence in their ability to perform specific learning tasks using an online learning system. A substantial body of research has demonstrated that online learning self-efficacy is a critical predictor of learning engagement. For instance, Wang X.-M. et al. (2022) found that learners with higher online learning self-efficacy exhibit greater focus and perseverance when faced with challenges, leading to higher levels of learning engagement. Kuo et al. (2021) pointed out that general online learning self-efficacy (e.g., mastering course content) contributes to behavioral and emotional engagement, while functional online learning self-efficacy (e.g., searching, registering, submitting assignments) promotes emotional and cognitive engagement.

For RNs, virtual simulation-based online learning presents distinct challenges. Particularly in China, RNs often face immense pressure due to a shortage of healthcare staff, high patient demands, and heavy workloads (Liu and Aungsuroch, 2019). This leads them to dedicate personal time (usually outside of regular working hours) to continuous learning and professional development. The dual pressures of time constraints and workload make it difficult for them to adopt structured self-regulation strategies, often relying on intrinsic motivation to drive their learning (Gkorezis et al., 2021). Achievement motivation, as an intrinsic driver of learners, encompasses the psychological tendencies of pursuing success and avoiding failure (McClelland, 2015), and directly influences learning behaviors and outcomes. Diseth and Kobbeltvedt (2010) argue that the higher a student's achievement motivation, the stronger their willingness to engage in learning tasks. Specifically, the motivation to pursue success positively predicts cognitive, behavioral, and emotional engagement, while the motivation to avoid failure negatively affects learning engagement. Alemayehu and Chen (2023) noted that in online learning environments, motivation is positively correlated with learning engagement, and motivation not only directly influences learning engagement but also exerts indirect effects through self-regulation. Additionally, a longitudinal study indicated that self-efficacy in middle school students can positively predict their learning motivation three semesters later (Alivernini and Lucidi, 2011). Therefore, achievement motivation may play a mediating role between online learning self-efficacy and learning engagement.

In addition, virtual simulation emphasizes an immersive learning experience, but the learning of RNs typically occurs during their off-duty hours, making them susceptible to distractions from external factors (e.g., family or work environments), which may divert their attention. Flow theory suggests that flow is described as the optimal engaging experience, characterized by intense focus, control, interest, and a balance between skills and challenges (Mahfouz et al., 2020). When learners enter a flow state, they become fully immersed in the learning task. This immersive experience promotes sustained cognitive involvement and emotional resonance, which further strengthens learning engagement (Goh and Yang, 2021). Özhan and Kocadere (2019) found that self-efficacy serves as a precursor to flow, predicting flow s over time. Park and Seo (2022) highlighted that in virtual learning environments, nursing student's self-regulated learning abilities, learning motivation, and self-efficacy are key factors influencing flows. Therefore, flow might be a mediating factor between online learning self-efficacy and learning engagement. Although flow and learning engagement both involve learners' focus and involvement in learning tasks, they differ conceptually and functionally. Flow refers to a short-term, immersive psychological state occurring during learning, characterized as a dynamic, process-oriented variable (Norsworthy et al., 2021). In contrast, learning engagement is a relatively stable disposition encompassing emotional, behavioral, and cognitive responses throughout the learning process (Martin and Borup, 2022). Accordingly, this study incorporates flow into the hypothesized model as a potential mediating process variable.

Flow is most likely to occur when individuals perceive a task as sufficiently challenging yet manageable using their existing skills (Mahfouz et al., 2020). Achievement motivation, as the intrinsic drive to set challenging goals and achieve skill growth, may influence the experience of flow by adjusting the challenge-skill balance. Several studies (Rachmatullah et al., 2021; Wang Y. et al., 2022) have also confirmed the positive relationship between achievement motivation and flow.

### 2.1 Theoretical framework

SCT, proposed by Bandura (2001), emphasizes that individual behavior results from the dynamic interaction between personal factors and behavioral patterns. In the context of online learning, self-efficacy is regarded as a proximal individual cognitive factor that significantly affects learning engagement. According to SCT, individuals with high self-efficacy are more motivated to overcome challenges and achieve their goals (Bandura, 2001). Within this framework, achievement motivation can be viewed as a concrete manifestation of the motivational processes stimulated by self-efficacy. Although flow experience is not explicitly addressed in SCT, it can be understood as a situational positive emotional state triggered by high self-efficacy and intrinsic motivation. When individuals perceive that their skills are well matched to the challenges at hand, they are more likely to enter a state of flow, which enhances both cognitive and emotional engagement.

Therefore, this study adopts SCT as the primary theoretical framework and incorporates flow theory as a complementary perspective to provide a more comprehensive explanation of how self-efficacy and motivation translate into sustained learning engagement.

**Figure 1:**
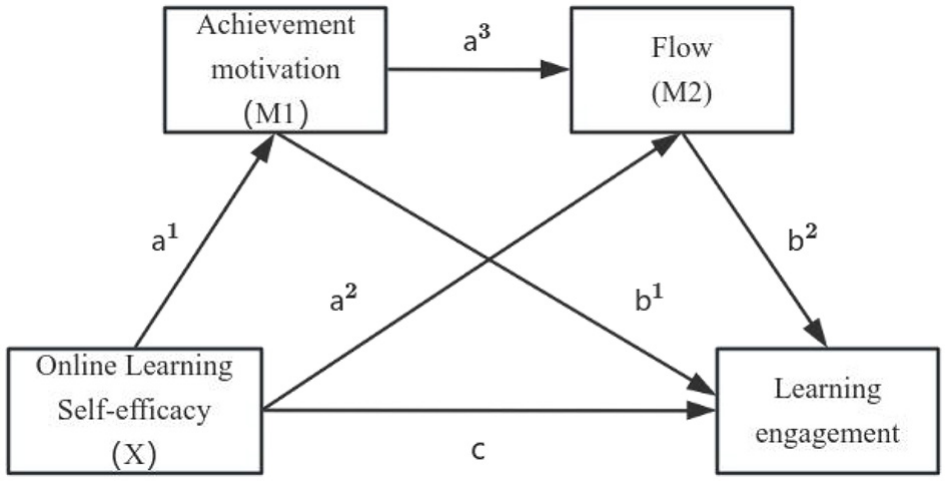
Hypothesis model.

Based on this framework, the present study constructs a chain mediation model to examine the mechanisms through which online learning self-efficacy influences learning engagement among registered nurses in a virtual simulation environment. The proposed theoretical model is presented in Figure 1, and the research hypotheses are as follows:

Hypothesis 1:

(c). Online learning self-efficacy is significantly and positively associated with learning engagement.

Hypothesis 2:

(a^1^b^1^). Achievement motivation significantly mediates the relationship between online learning self-efficacy and learning engagement.

Hypothesis 3:

(a^2^b^2^). Flow significantly mediates the relationship between online learning self-efficacy and learning engagement.

Hypothesis 4:

(a^1^a^3^b^2^). Achievement motivation and flow play chain-mediating roles between online learning self-efficacy and learning engagement.

## 3 Methods

### 3.1 Design and sample

A descriptive, cross-sectional design was adopted in the present study. A convenience sampling method was used to select participants from two comprehensive hospitals in Sichuan Province, China. The inclusion criteria were: (1) possession of a valid nursing qualification certificate; (2) informed consent and voluntary participation; and (3) normal cognitive function and no communication barriers. The exclusion criteria were: (1) interns or nurses not formally employed at the study hospitals (e.g., visiting trainees from other institutions); and (2) RNs on leave or not currently working. The study was conducted with the consent of hospital nursing administrators and participants, following the principles of voluntary participation, anonymity, and data confidentiality, in accordance with the ethical guidelines of the Declaration of Helsinki (World Medical Association, 2024).

A sample size calculation was performed using the formula n = ${\left(\mu_{\frac{\alpha \;}{2}}\sigma /\delta \right)}^{2}$, with the significance level α set at 0.05 ($\mu_{\frac{\alpha }{2}}\;=$1.96), and the allowable error δ set at 2.0. Based on a pilot survey involving 30 clinical nurses, the standard deviation of the Achievement Motivation Scale (σ = 20.92) was used as the reference. Considering a 20% expected attrition rate, the required minimum sample size was 525. In this study, a total of 689 questionnaires were distributed and collected. After excluding invalid responses (e.g., completion time <60 seconds, contradictory answers, or overly patterned responses), 657 valid questionnaires were included for analysis, resulting in an effective response rate of 95.36%, which met the estimated sample size requirement.

### 3.2 Procedures

This study used the 'Snake Bite Treatment Thinking—Using the Bungarus as an Example' virtual simulation course (https://www.ilab-x.com/) as the teaching material. Developed from real clinical cases, the course incorporated four immersive scenarios—pre-hospital care, emergency resuscitation room, ICU, and general emergency ward—that simulated the complete progression of Bungarus envenomation. All participants underwent the same standardized 3-week training program, ensuring uniformity in the course content provided. To ensure the flexibility and feasibility of the training, no centralized or scheduled sessions were arranged. Participants were allowed to complete the learning tasks independently during their spare time (e.g., in the evenings or on weekends), based on their individual work schedules.

Within the virtual simulation, participants engaged in virtual patient observation, treatment, emergency care, nursing procedures, health education, and communication through human-computer interaction, menu-based operations, and dialogue-based decision-making. Each scenario was designed with specific nursing tasks, covering both fundamental and advanced nursing interventions, aimed at enhancing participants' clinical knowledge, basic skills, emergency response abilities, and clinical reasoning. For example, in the pre-hospital scenario, participants simulated the role of a triage nurse, communicating with the patient's family to collect medical history and providing instructions for initial first aid. In the emergency resuscitation room scenario, participants utilized multi-parameter monitors to assess vital signs and performed 13 critical emergency procedures, such as cardiopulmonary resuscitation (CPR) and bag-valve mask (BVM) ventilation.

Data were collected via an online survey platform (http://www.sojump.com) 1 week after the course concluded. The platform generated a poster with a QR code, which was distributed to hospital nursing administrators by the researchers and displayed during a centralized meeting. Participants were invited to scan the QR code and complete the survey. Standardized instructions were provided during the meeting, clearly outlining the study's purpose, ensuring confidentiality, and offering uniform explanations in case participants had any questions. To ensure data completeness and validity, all survey questions were mandatory, and each IP address was allowed only one submission to prevent duplicate responses.

### 3.3 Instrument

#### 3.3.1 Demographics

A self-developed general information questionnaire was used to collect demographic data, including gender, age, education level, hospital level, years of work experience, and professional title.

#### 3.3.2 Adult online learning self-efficacy scale

This scale was developed by Li et al. (2015) and consists of three dimensions: Learning Capacity (seven items), Willingness to Learn (seven items), and Learning Techniques (nine items), totaling twenty three items. Responses are measured using a 5-point Likert scale, ranging from 1 (Strongly Disagree) to 5 (Strongly Agree). The total score ranges from 23 to 115, with higher scores indicating greater self-efficacy. Exploratory factor analysis demonstrated that the 3-factor (Learning Capacity, Willingness to Learn, and Learning Techniques) structure of the scale explained 73.33% of the total variance, and the Cronbach's α coefficient was 0.966 (Li et al., 2015). In the present study, the Cronbach's α coefficient was 0.932.

#### 3.3.3 Achievement motivation scale (AMS)

The scale was originally developed by Gjesme et al. and was later adapted into Chinese by Ye (1992). It comprises two dimensions: Motivation to Succeed (MS) with fifteen items and Motives to Avoid Failure (MAF) with fifteen items, totaling thirty items. Responses are assessed using a 5-point Likert scale, ranging from 1 (Strongly Disagree) to 5 (Strongly Agree). The total score is calculated as the difference between the Motivation to Succeed score and the Motives to Avoid Failure score, with higher scores indicating stronger achievement motivation. The Cronbach's α coefficient for the original scale was 0.867 (Ye, 1992). In the current study, the Cronbach's α coefficient was 0.831.

#### 3.3.4 Education flow-2 scale (edu flow-2)

The Education Flow-2 Scale was developed by Heutte et al. (2021) and includes four dimensions: Cognitive Control (3 items), Immersion and Time Transformation (three items), Loss of Self-Consciousness (three items), and Autotelic Experience—Wellbeing Provided by the Activity (three items), totaling twelve items. Responses are measured on a 7-point Likert scale, ranging from 1 (Strongly Disagree) to 7 (totally agree), with higher scores reflecting a better flow. Confirmatory factor analysis indicated a good fit for the model (CFI = 0.99, TLI = 0.99) (Heutte et al., 2021). In this study, the Cronbach's α coefficient was 0.915.

#### 3.3.5 Utrecht work engagement scale-student (UWES-s)

The Utrecht Work Engagement Scale for Students (UWES-S) was developed by Schaufeli et al. (2002) and later revised by Fu (2022). It includes three dimensions: Vigor (six items), Dedication (five items), and Absorption (five items), totaling sixteen items. Responses are recorded on a 5-point Likert scale, ranging from 1 (Strongly Disagree) to 5 (Strongly Agree), with higher scores indicating greater engagement in online learning. The Cronbach's α coefficient for the scale ranges from 0.765 to 0.813 (Fu, 2022). In this study, the Cronbach's α coefficient was 0.899.

### 3.4 Data analysis

Data analyses were performed using SPSS V.29.0 and AMOS V.24.0 software. Categorical data were described by frequencies and percentages, and continuous data by means with Standard Deviations (SD). Pearson correlation analysis was conducted to examine the relationships between online learning self-efficacy, achievement motivation, flow, and learning engagement among RNs. Multicollinearity was tested using tolerance and Variance Inflation Factor (VIF), and condition indices. A structural equation model was formulated and verified using Amos V.24.0, incorporating a bootstrap methodology (executed 5,000 times) to assess mediation effects (95%CI, *P* < 0.05). The model's fitness was evaluated employing various indices, including chi-square/degree of freedom ratio (χ2/*df* ), root mean square error of approximation (RMSEA), the Adjusted Goodness-Of-Fit Index (AGFI), the Comparative Fit Index (CFI), the Goodness of Fit Index (GFI), the Incremental Fit Index (IFI), and Tucker Lewis index (TLI). A two-tailed *P* < 0.05 was regarded as statistical significance.

### 3.5 Ethical considerations

This study was conducted following approval from the Ethics Committee of Suining Central Hospital (NO. KYLLKS20240105). Prior to participation, all participants provided informed consent and were informed of their right to withdraw from the study at any stage without consequence. The study adhered to ethical principles, ensuring the anonymity and confidentiality of all participants.

## 4 Result

### 4.1 Common method biases test

To address potential common method bias from self-reported data, anonymity and confidentiality were emphasized, and reverse questions were included. The Harman single-factor test was used statistically to test for common method bias. The results revealed the extraction of twelve factors with eigenvalues greater than 1, with the largest factor explaining 26.4% of the variance (less than 40.0%). Tolerance values ranged from 0.683 to 0.757 (< 1.00), and VIF ranged from 1.321 to 1.531 (< 10.00). Additionally, condition indices ranged from 3.239 to 12.801 (<30), indicating no significant multicollinearity issues (Podsakoff et al., 2003).

### 4.2 Participants' general characteristics

In the 657 valid questionnaires, respondents' ages ranged from 22 to 50 years (mean age: 30.27 ± 2.00). Most were female (*n* = 616, 93.8 %). Table 1 presents the additional characteristics. The total score was 75.02 ± 16.35 for online learning self-efficacy, 9.73 ± 21.45 for achievement motivation, 59.66 ± 14.66 for flow, and 56.72 ± 10.41 for learning engagement, as presented in Table 2.

**Table 1:** Sample characteristics (*N* = 657).

**Characteristics**	***N***	**%**
**Gender**		
Male	41	6.2
Female	616	93.8
**Education level**		
Associate degree	374	56.9
Bachelor's degree and above	283	43.1
**Hospital level**		
Level III	323	49.2
Level II	334	50.8
**Years of work experience**		
≤5years	295	44.9
6-10years	232	35.3
11-15years	82	12.5
>15years	48	7.3
**Professional title**		
Nurse	277	42.2
Nurse practitioner	255	38.8
Supervisor nurse	103	15.7
Chief nurse	22	3.3

**Table 2:** Descriptive statistics of the four variables (*N* = 657).

**Variable**	**Min**	**Max**	**Mean**	***SD***
Online learning self-efficacy	23	111	75.02	16.35
Learning capacity	7	35	24.31	5.97
Willingness to learn	7	35	23.46	6.26
Learning techniques	9	44	27.25	7.83
Achievement motivation	−56	52	9.73	21.45
Motivation to succeed	15	72	48.42	12.78
Motives to avoid failure	18	75	38.69	12.48
Flow	15	83	59.66	14.66
Cognitive control	3	21	14.75	4.50
Immersion and time transformation	3	21	14.61	4.52
Loss of self-consciousness	3	21	14.89	4.52
Autotelic Experience—Wellbeing Provided by the Activity	3	21	15.41	4.34
Learning engagement	24	79	56.72	10.41
Vigor	6	30	21.05	5.14
Dedication	6	25	17.90	4.04
Absorption	6	25	17.77	4.25

SD,Standard Deviation.

### 4.3 Bivariate correlations of the measured variables

As presented in Table 3, online learning self-efficacy was positively correlated with achievement motivation, flow, and learning engagement (*r* = 0.408, 0.530, 0.498, *P* < 0.01). Achievement motivation was significantly related to flow and learning engagement (*r* = 0.450, 0.424, *P* < 0.01). Flow was also related to learning engagement (*r* = 0.550, *P* < 0.01).

**Table 3:** Bivariate correlations of the measured variables (*N* = 657).

**Variables**	**1**	**2**	**3**	**4**	**5**	**6**	**7**	**8**	**9**	**10**	**11**	**12**	**13**	**14**	**15**	**16**
1.Online learning self-efficacy	1															
2.Learning ability	0.756^**^	1														
3.Learning will	0.833^**^	0.498^**^	1													
4.Learning skills	0.845^**^	0.418^**^	0.559^**^	1												
5.Achievement motivation	0.408^**^	0.335^**^	0.325^**^	0.338^**^	1											
6.Motivation to succeed	0.329^**^	0.254^**^	0.249^**^	0.294^**^	0.853^**^	1										
7.Motives to avoid failure	−0.365^**^	−0.316^**^	−0.302^**^	−0.279^**^	−0.845^**^	−0.443^**^	1									
8.Flow	0.530^**^	0.411^**^	0.436^**^	0.445^**^	0.450^**^	0.353^**^	−0.412^**^	1								
9.Cognitive control	0.417^**^	0.345^**^	0.330^**^	0.344^**^	0.368^**^	0.281^**^	−0.345^**^	0.806^**^	1							
10.Immersion and time transformation	0.457^**^	0.316^**^	0.389^**^	0.403^**^	0.392^**^	0.321^**^	−0.345^**^	0.804^**^	0.502^**^	1						
11.Loss of self-consciousness	0.414^**^	0.346^**^	0.332^**^	0.336^**^	0.356^**^	0.285^**^	−0.321^**^	0.824^**^	0.547^**^	0.549^**^	1					
12.Autotelic Experience— Wellbeing Provided by the Activity	0.449^**^	0.342^**^	0.377^**^	0.376^**^	0.359^**^	0.269^**^	−0.341^**^	0.843^**^	0.590^**^	0.581^**^	0.602^**^	1				
13.Learning engagement	0.498^**^	0.401^**^	0.403^**^	0.412^**^	0.424^**^	0.312^**^	−0.409^**^	0.550^**^	0.426^**^	0.448^**^	0.453^**^	0.476^**^	1			
14.Vigor	0.416^**^	0.330^**^	0.340^**^	0.346^**^	0.346^**^	0.254^**^	−0.335^**^	0.468^**^	0.376^**^	0.379^**^	0.395^**^	0.384^**^	0.784^**^	1		
15.Dedication	0.360^**^	0.288^**^	0.284^**^	0.304^**^	0.320^**^	0.230^**^	−0.314^**^	0.390^**^	0.306^**^	0.308^**^	0.303^**^	0.362^**^	0.768^**^	0.373^**^	1	
16.Absorption	0.375^**^	0.309^**^	0.307^**^	0.302^**^	0.314^**^	0.237^**^	−0.298^**^	0.410^**^	0.295^**^	0.347^**^	0.344^**^	0.358^**^	0.770^**^	0.355^**^	0.478^**^	1

^**^P < 0.01.

### 4.4 Structural equation model test

The theoretical model fit indices were set as follows: χ^2^*/df* < 3, *RMSEA* < 0.05, AGFI > 0.9, *CFI* > 0.90, *GFI* > 0.90, *IFI* > 0.90, and *TLI* > 0.90 (Kline, 2016). In this study, the obtained model fit indices were χ^2^*/df* = 1.467, *RMSEA* = 0.027, *AGFI* = 0.971, *CFI* = 0.991, *GFI* = 0.982, *IFI* = 0.991, and *TLI* = 0.988, all of which met the theoretical criteria. These results confirm that the hypothesized path model exhibited an acceptable fit. The detailed fit indices are presented in Table 4.

**Table 4:** Structure equation modeling fit indices.

**Fitness**	***χ^2^/df***	***RMSEA***	***SRMR***	***CFI***	***GFI***	***IFI***	***TLI***
Acceptable values	<3	<0.05	>0.90	>0.90	>0.90	>0.90	>0.90
Mediation model	1.467	0.027	0.971	0.991	0.982	0.991	0.988

χ^2^/df, Chi-square divided by degrees of freedom; RMSEA,Root Mean Square Error of Approximation; AGF, the Adjusted Goodness Of Fit Index; CFI, Comparative Fit Index; GFI, Goodness of Fit Index; AGFI, Adjusted Goodness of Fit Index; IFI, Incremental Fit Index; TLI, Tucker-Lewis Index.

### 4.5 Effects of structural equation model

**Figure 2:**
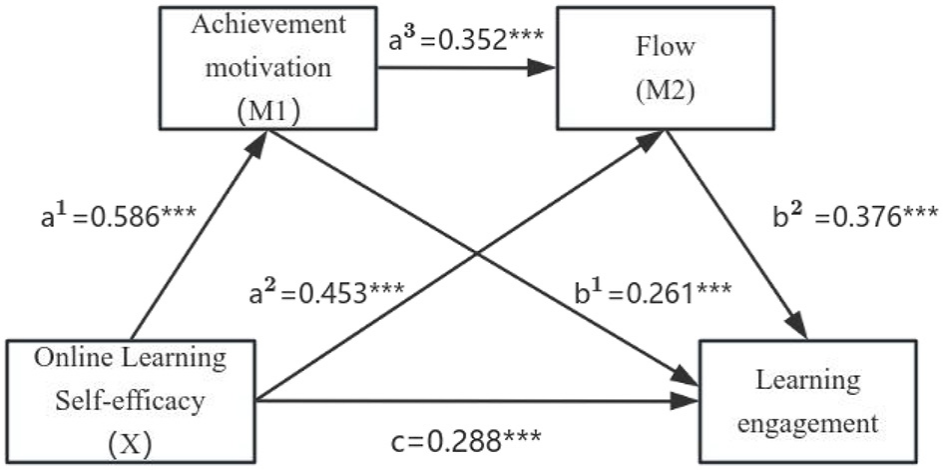
Final path diagram of the mediating effects of achievement motivation and flow on the relationship between online learning self-efficacy and learning engagement. ***P < 0.001.

The results indicated that Online learning self-efficacy demonstrated statistically significant direct effects on Achievement motivation (0.586), Flow (0.453), and Learning engagement (0.288). Additionally, Achievement motivation had a direct positive effect on Flow (0.352) and Learning engagement (0.261), while flow further contributed to learning engagement (0.376). The *R*^2^ value for Learning engagement was 0.644, indicating that 64.4% of the variance in Learning engagement was explained by the predictors in the model. Furthermore, the bootstrap analysis confirmed that Achievement motivation (0.586^*^0.261 = 0.153) and Flow (0.586^*^0.352^*^0.376 = 0.077) significantly mediated the relationship between Online learning self-efficacy and Learning engagement. Although Online learning self-efficacy exerted a direct effect, its impact was also transmitted indirectly through Achievement motivation and Flow. The total effect of Online learning self-efficacy on Learning engagement was 0.689 (0.288 + 0.586^*^0.261^*^0.586^*^0.352^*^0.376), with mediation accounting for 58.1% of the total effect. A summary of the mediation effects between online learning self-efficacy and learning engagement is provided in Table 5. The path diagram for the model is illustrated in Figure 2.

**Table 5:** The standardized total, direct, and indirect effects of online learning self-efficacy on learning engagement.

**Model Pathway**	**B**	**SE**	**95%CI**	**Percent (%)**
			**Lower**	**Upper**	
Total effect	0.689	0.058	0.568	0.796	100
Direct effect	0.288	0.087	0.124	0.470	41.9
Indirect effect	0.400	0.060	0.287	0.518	58.1
a^1^b^1^:OLSE → AM → LE	0.153	0.063	0.029	0.272	22.2
a^2^b^2^:OLSE → F → LE	0.170	0.046	0.094	0.272	24.7
a^1^a^3^b^2^:OLSE → AM → F → LE	0.077	0.028	0.037	0.151	11.2

OLSE, Online Learning Self-Efficacy;AM, Achievement Motivation;F, Flow;LE, Learning Engagement;B Standardized Coefficients;SE, Standard Error;95%CI, Percentile 95% confidence interval.

## 5 Discussion

The purpose of this study was to explore the relationships among online learning self-efficacy, achievement motivation, flow, and learning engagement among RNs, as well as to examine the mediating roles of achievement motivation and flow between online learning self-efficacy and learning engagement. The results indicated that: (1) there are significant positive correlations among online learning self-efficacy, achievement motivation, flow, and learning engagement; (2) achievement motivation and flow partially mediate the relationship between online learning self-efficacy and learning engagement; and (3) achievement motivation and flow exhibit significant chain mediation effects between online learning self-efficacy and learning engagement.

The findings of this study revealed that the average score for learning engagement among RNs was 3.54 ± 0.65, indicating a moderate level. This result is similar to the findings of Zhang et al. (2024), but lower than those observed among nursing undergraduates participating in online learning (Huang et al., 2023; Liao et al., 2024). This discrepancy may be attributed to differences in professional stages. Nursing students are primarily motivated by the pursuit of academic achievement and expectations for future career development (Capelle et al., 2022), whereas RNs are more inclined to meet practical work demands and less proactive in focusing on their own continuous development. Additionally, nursing students often receive more support from instructors, enjoy a favorable educational environment, and have relatively ample time for learning (An et al., 2022). In contrast, RNs frequently face heavy workloads and professional pressures, which result in limited time and energy for self-directed learning (Broetje et al., 2020). Furthermore, the relative scarcity of training opportunities and the lack of guidance and support may diminish their level of learning engagement. Therefore, nursing managers are encouraged to optimize workload arrangements, reduce excessive job stress, allocate resources more effectively, and provide flexible, efficient learning opportunities. These strategies can stimulate nurses' interest and initiative in learning, thereby improving their engagement levels.

This study found a significant positive correlation between online learning self-efficacy and learning engagement among RNs. This result aligns with the studies by Dai (2024) and Yokoyama (2024), which suggest that individuals with higher online learning self-efficacy exhibit greater engagement in learning activities. According to Bandura's self-efficacy theory (Bandura, 1977), self-efficacy influences individuals' levels of effort, perseverance, and emotional regulation, thereby having a substantial impact on behavioral performance. In an online learning environment, RNs with low self-efficacy tend to underestimate their abilities in complex learning situations, making them more likely to choose to quit or withdraw, and struggle to effectively resolve the problems they encounter. Conversely, RNs with higher self-efficacy typically exhibit greater confidence and persistence when facing learning tasks. They proactively address challenges, employ effective learning strategies, and actively seek feedback and engage in self-reflection, which enhances their learning engagement. Moreover, RNs with high self-efficacy are better able to maintain emotional stability when encountering difficulties, avoiding anxiety and frustration, and continuing their learning endeavors (Wang X.-M. et al., 2022). Based on these findings, nursing managers are encouraged to enhance RNs' self-efficacy by embedding progressive tasks and immediate performance feedback into training programs. Additionally, establishing peer support networks and showcasing positive role models can offer vicarious learning experiences. Contextualized emotional regulation strategies should also be considered to help RNs manage learning-related anxiety and enhance their engagement.

Another finding of this study is that achievement motivation significantly mediates the relationship between online learning self-efficacy and learning engagement. According to self-determination theory, self-efficacy enhances intrinsic motivation by fulfilling the three basic needs of autonomy, competence, and relatedness (Howard et al., 2021). Relevant research indicates that intrinsic or internalized motivation, as opposed to extrinsic motivation, more effectively drives learners to exhibit higher levels of engagement in learning activities (Chan et al., 2023). In this study, achievement motivation, as an intrinsic motivator, is influenced by the flexibility, challenge, and interactivity of virtual simulation platforms. Specifically, the flexibility of virtual simulation platforms allows RNs to autonomously adjust their learning pace, enhancing their sense of autonomy; the challenge of tasks increases their sense of competence; and interactivity fosters a sense of relatedness among RNs. These factors effectively satisfy the three basic needs outlined in self-determination theory, thereby promoting RNs' pursuit of higher goals and increasing their learning engagement. Additionally, RNs with high online learning self-efficacy experience a sense of achievement after successfully solving problems, which stimulates their enthusiasm for learning. This finding is consistent with and extends the studies of Yokoyama (2019), thereby deepening the understanding of the multifaceted impact of achievement motivation in nursing education. In response, nursing managers should guide RNs in setting personal learning goals and plans to foster autonomy and self-direction. Integrating reward mechanisms, such as learning points exchange, recognition, or promotion opportunities, may stimulate achievement motivation and enhance proactive learning.

Moreover, the study elucidates the mediating role of flow in the relationship between online learning self-efficacy and learning engagement, consistent with prior studies (Yeh et al., 2019; Yoo and Kim, 2018). This finding aligns with Csikszentmihalyi's (1990) flow theory, which posits that individuals are more likely to enter a flow state when their skill level matches the challenge of the task. RNs with high online learning self-efficacy are better able to recognize and adjust this matching relationship, thereby facilitating their entry into a flow state. In a flow state, RNs can concentrate their attention, minimize external distractions, and enhance their immersion in learning, thereby increasing their level of learning engagement. Nursing administrators can improve the design of learning tasks by incorporating tiered difficulty levels, allowing RNs to select content based on their capabilities and progress. Ensuring that tasks strike a balance between challenge and skill level will enhance learners' sense of control, autonomy, and immersion, fostering deeper engagement.

One key finding of this study is that the mediation effects account for 58.1% of the total effect. This result highlights the importance of not only enhancing online learning self-efficacy, but also cultivating RNs' internal drive for excellence and immersive learning experiences to ultimately boost engagement. Achievement motivation drives RNs to pursue excellence and improvement in learning. This strong goal orientation increases their likelihood of maintaining focused attention and entering a flow state, thereby enhancing learning engagement. Therefore, nursing managers are encouraged to integrate ideological and political education into continuing training programs. This approach helps reinforce nurses' professional responsibility, providing value support for achievement motivation. Managers should also strengthen the connection between learning behaviors, professional development, and societal contributions to sustain long-term goal orientation. Moreover, assisting nurses in setting clear and appropriately challenging learning goals, along with providing timely feedback, can effectively trigger flow experiences and enhance online learning engagement.

## 6 Limitation and research recommendations

In this study, several limitations should be noted. First, the survey was conducted among RNs from two hospitals, which may limit the generalizability of the findings. Therefore, future research should continue to explore and expand the sample size. Second, the study employed a cross-sectional design, which revealed relationships among variables but restricted causal inferences. Longitudinal studies are necessary to investigate the causal relationships of these variables over time. Third, the study relied on self-reported data, which may be subject to social desirability bias and recall bias. Future studies should adopt a mixed-methods approach, integrating both quantitative and qualitative methods to enhance the reliability and accuracy of the results. Finally, grounded in SCT, this study focused primarily on individual psychological mechanisms influencing learning engagement. Other potentially relevant factors—such as contextual elements (e.g., learning time, content interest, interface design) and broader influences (e.g., organizational support, policy changes, career development opportunities)—were not included. Future research may consider incorporating these variables to build a more comprehensive explanatory model.

## 7 Conclusion

This study demonstrated that online learning self-efficacy has a significant impact on learning engagement among RNs, and this relationship is strengthened by the mediating roles of achievement motivation and flow. The findings offer a new perspective on online learning for RNs, emphasizing the critical roles of self-efficacy, achievement motivation, and flow in learning engagement. Based on these results, nursing managers can implement intervention strategies to cultivate RNs' online learning self-efficacy, stimulate their achievement motivation, and optimize learning designs to enhance flow, thereby promoting higher levels of learning engagement among RNs. These findings provide theoretical support for improving the effectiveness of RNs' online learning and offer practical guidance for future nursing education practices and ongoing professional development.

## Data Availability

The raw data supporting the conclusions of this article will be made available by the authors, without undue reservation.
